# The early learning curve of the bipolar enucleation of the prostate: a multicenter cohort study

**DOI:** 10.1007/s00345-024-05183-y

**Published:** 2024-08-08

**Authors:** Christian Ramesmayer, Susanne Deininger, Nikolaos Pyrgidis, Lukas Lusuardi, Thomas Kunit, Maximilian Pallauf, Manuela Sieberer, Martin Drerup, Paolo Fontanella, David Oswald, Thomas RW Hermann, Evangelos N. Symeonidis, Dimitrios Memmos, Petros Sountoulides

**Affiliations:** 1 Department of Urology and Andrology, Paracelsus Medical University, Müllner Hauptstraße 48, Salzburg, 5020 Austria; 2 Department of Urology, University Hospital, LMU Munich, Munich-Marchioninistr. 15, 81377 Munich, Germany; 3 Department of Urology, Hospital Brothers of St. John, Kajetanerplatz 1, Salzburg, Austria; 4 Department of Urology, Spital Thurgau AG, Kantonspital Frauenfeld, Frauenfeld, Switzerland; 5 Urology Stellenbosch University, Western Cape, South Africa; 6 Hannover Medical Scholl, MHH Carl Neuberg Str. 1, 30625 Hannover, Germany; 7 1st Department of Urology, Aristotle University of Thessaloniki, Thessaloniki, Greece

**Keywords:** Benign prostatic hyperplasia, BipolEp, Learning curve, Enucleation, BPH

## Abstract

**Objectives:**

To evaluate the early learning curve of BipolEP (Bipolar Enucleation of the Prostate).

**Subjects/patients and methods:**

We conducted a retrospective, multicenter analysis of surgical and functional outcomes of patients treated with BipolEp for BPO (benign prostatic obstruction). We evaluated the first 20 cases of BipolEp performed by four different surgeons in three different countries. The following baseline parameters were obtained: age, IPSS, indwelling catheter, transrectal measured prostate volume, post void residual volume (PVR) and uroflowmetry. The learning curve was analysed based on perioperative parameters and the influence of perioperative parameters was correlated with the sequence of BipolEp cases.

**Results:**

84 BipolEp operations performed by 4 different surgeons in their early learning curve were studied. Mean prostate volume was 75 ml, 39% of cases had an indwelling catheter and the average operating time was 101 min. Three out of four surgeons performed at least 50% of successful operations according to Trifecta (complete enucleation and morcellation < 90 min., no conversion to TUR-P). Conversion rate to TURP was 11.9% in total which however was driven by a single surgeon with an almost 50% conversion rate. Mean enucleated prostate was 33.3 gr (18-54.5). Intraoperative complications and reported stress incontinence ranged from 0 to 38.1%. At six-weeks review, the IPPS improved by 12.5 (8–16) points and Qmax by 208% (109.8-266.7). Uroflowmetry outcomes correlated with the sequence of cases with a linear improvement during 20 consecutive cases (*p* = 0.018) in all centres. Major complications (Clavien Dindo ≥ 3) were rare (4.8%) and comparable between the groups.

**Conclusion:**

Surgeons starting to learn BipolEp can expect to be able to achieve a linear improvement in Uroflow at the six-week postoperative evaluation after 20 consecutive cases. BipolEp can be successfully performed during the early learning curve with an acceptable rate of conversion to standard TUR-P.

## Introduction

Anatomical endoscopic enucleation of the prostate techniques (AEEP) (HoLEP, GreenlEp, BipolEp, ThuLEP, ThuFLEP) have been established as safe and feasible techniques for treatment of benign prostatic obstruction even compared to standard Transurethral resection of the prostate (TUR-P) [1] especially in large glands. In 1998 the first endoscopic enucleation technique, the Holmium laser enucleation of the prostate (HoLEP), showed similar effectiveness compared to open prostatectomy (OP) yet with lower morbidity [2]. When compared to TUR-P, AEEP has shown certain significant benefits, namely a lower hemoglobin drop, much lower transfusion rate and a shorter catheter time [3]. Despite these advantages, AEEP is still not the standard procedure for BPO in many urological departments. The learning curve seems to be a major limitation for wider adoption of this technique [4, 5]. The Holmium laser enucleation of the prostate (HoLEP) with its prolonged operating time and difficult-to-learn technique was prospectively evaluated by Robert et al. in 2016 [6] only to show that half of the centres initially aiming of adopting HoLEP abandoned this technique due to the steep learning curve. According to two study groups, a minimum of fifty procedures will need to be performed in order to overcome the learning curve of HolEP [6, 7]. An alternative to HoLEP was presented in 2006 with BipolEP showing similar results [8]. One of the advantages of BipolEP is the easy conversion to standard bipolar TUR-P within minutes which allows the surgeon to learn the technique step-by-step without compromising the patients outcomes. One study evaluating the learning curve of BipolEP concluded that after 30 cases the likelihood of conversion to TUR-P rate is reduced [9]. To our knowledge, there are no other studies evaluating the learning curve of BipolEP among surgeons relatively naïve to the enucleation technique. We therefore aimed to analyse this issue in a multicentre, retrospective study.

## Subjects/patients and methods

### Study protocol

An ethical approval was obtained from the local ethical committee to conduct the present retrospective cohort study. In this multicenter study, we included all patients with BPO, who were treated with BipolEp at three different departments (Paracelsus Medical University, Salzburg; Spital Thurgau AG, Frauenfeld; Aristotle University of Thessaloniki) by 4 surgeons during their very early learning curve with the procedure. All surgeon have had significant experience in endoscopic urology TUR-P in particular. The following baseline parameters were obtained: age, indwelling catheter preoperatively, retention volume, transrectal measured prostate volume, post void residual volume (PVR), international prostate symptom score (IPSS) and Qmax. Patient were excluded from this study if there was any previous prostatic surgery or confirmed prostate cancer. All patients gave written informed consent to participate.

### BipolEP procedure and outcomes

All participants were treated with BipolEP by four different surgeons at 3 different countries. All surgeons were senior urologists and had significant experience to transurethral surgery including TUR-P but had very limited experience in BipolEP. In fact these were the first 20 cases of BipolEP performed unsupervised without the presence of a mentor following a short initial mentorship period.

All BipolEP cases were performed using Storz equipment (Karl Storz Endoskope, Tuttlingen, Germany) including 26 F continuous flow bipolar resectoscope, the specially designed Thomas Herrmann enucleation loop and the Storz (Karl Storz Endoskope, Tuttlingen, Germany) morcellator. As all surgeons were performing their first bipolep cases unsupervised, the AEEP technique used was the two or three lobe technique as en-bloc enucleation is more technically demanding. With regard to treatment of the sphincter the technique was not standardized and left to the individual surgeon’s discretion whether or not to start the procedure with early release of the sphincter.

A successful BipolEp was defined as complete enucleation and morcellation without conversion to TUR-P (Trifecta). All patients were evaluated 6 weeks and at 3 months after the operation.

Perioperative data were available for the following parameters: number of lobes enucleated (1, 2 and/or middle lobe), enucleation time, conversion to TUR-P, morcellation time, total operating time, enucleated volume and percentage of enucleated adenoma/total prostate volume, trial without catheter (TWOC) day and discharge day, intraoperative complications (bladder injury during morcellation, morcellator malfunction etc.) Patients had blood taken for Hemoglobin and biochemistry on the first post operative day, therefore additional data were available on Hb drop, need for transfusions and sodium levels.

During the first patient review at 6 weeks and also the 2nd follow up visit at 6 months the following data were gathered: IPSS, PSA and PSA reduction %, uroflowmetry including Qmax, voided volume and PVR and percentage of readmission for BipolEP related complications (urethral stricture, hematuria, etc.)

### Statistical analysis

All continuous variables were assessed for normality with the Kolmogorov-Smirnov test and comparisons among the groups were performed with the corresponding statistical tests. Accordingly, categorical variables were compared with the exact chi-square test. The effect of the learning curve on perioperative and follow-up outcomes was evaluated with logistic and linear regression models. All statistical analyses were performed using the R statistical software package, version 4.0.5.

## Results

We included a total 84 BipolEP operations performed by 4 surgeons at three different centers. No surgeon had any prior experience with BipolEp apart form a short mentee period and all cases were performed with the surgeon unsupervised. The median patient age was 72.5 (IQR: 67-77.6) years, the median IPSS 19 (IQR: 15–22) points and the median Qmax 6.5 (IQR: 5.1–8.8) mL/sec. A total of 64 (75%) patients were on anticoagulants and 50 (61%) had preoperatively an indwelling catheter for retention. No statistically significant differences were observed in the baseline characteristics among the patients operated by each surgeon. All values are summarized in Table 1.

**Table 1:** Baseline characteristics. Values are presented as mean ± standard deviation or median with interquartile range and n (%)

	Total	1 surgeon	2 surgeon	3 surgeon	4 surgeon	*p*-value
Number of cases	84	17	20	25	22	
Age (yr)	72.5 (67.4–77.6)	71.5 (66.3–81.4)	74.3 (68-75.6)	73.6 (69.3–77.1)	70.6 (67.3–78.7)	0.990
IPSS	19 (15–22)	14 (8–16)	18 (16–20)	25 (15–25)	19 (16.5–20)	0.077
Qmax (mL/sec)	6.5 (5.1–8.8)	n/a	4.5 (4.1–4.8)	6.3 (5.4–7.5)	6.6 (6-9.2)	0.096
PSA (ng/ml)	**4.6 (2-9.3)**	**8.3 (5.6–11.9)**	**6.2 (4.1–12.7)**	**3.7 (2.4–8.1)**	**1.7 (0.9–3.1)**	**< 0.001**
PV (gr)	**75 (57–93)**	**91 (77–105)**	**110 (83–130)**	**60 (50–75)**	**63 (54–75)**	**< 0.001**
BPH medication – n (%)	**63 (77.8)**	**9 (60)**	**9 (45)**	**24 (100)**	**21 (95.5)**	**< 0.001**
Anticoagulants – n (%)	20 (25.3)	4 (26.7)	8 (42.1)	5 (21.7)	3 (13.6)	0.209
Indwelling catheter – n (%)	**33 (39.3)**	**8 (47.1)**	**13 (65)**	**6 (24)**	**6 (27.3)**	**0.012**

IPSS = International Prostate symptom score; PV = Prostate volume

The median operating time was 101 (IQR: 75, 122) minutes. Overall, the enucleated prostate volume was 33 (IQR: 18–55) grams, the postoperative Hb drop was 1 g/dl (IQR: 0.5–1.8) and the median hospital stay was 3 (IQR: 2–4) days. The overall conversion rate was 11.9% as one surgeon had to convert to TUR-P in 9 out of his 17 whereas the other three surgeons had one conversion in 67 cases. The median catheter dwell time postoperatively was 2 (2–3) days. In overall, 60% of all operations were successfully performed according to the Trifecta-criteria [6]. One surgeon performed 17.6% (3/17) of the operations according to Trifecta. Intraoperative complications ranged from 0 to 38.1% depending on the surgeon performing the operation. All perioperative data are presented in Table 2.

**Table 2:** Perioperative data. Values are presented as mean ± standard deviation or median with interquartile range and n (%)

	Total	1	2	3	4	*p*-value
Number of cases	84	17	20	25	22	
Operative time (min)	101 (75–122)	124 (98–143)	103.5 (74.5-121.5)	96 (76–110)	95 (80–110)	0.083
**Enucleated volume (g)**	**33.3 (18-54.5)**	**46 (30.8–56)**	**60 (34-87.6)**	**15.5 (11.5–27.5)**	**32 (21–43)**	**< 0.001**
Hb drop (g/dl)	1 (0.5–1.8)	0.9 (0.4–2.2)	1.3 (1-2.7)	0.8 (0.3–1.5)	1 (0.5–1.5)	0.183
**Hospitalisation (d)**	**3 (2–4)**	**4 (3–6)**	**4 (3-4.5)**	**3 (3–4)**	**2 (2–2)**	**< 0.001**
Clavien Dindo Complications, n (%)						0.105
1	10 (12)	1 (5.9)	1 (5)	0	8 (38.1)	
4	1 (1.2)	1 (5.9)	0	0	0	
Blood transfusion – no. (%)	5 (6.1)	2 (12.5)	1 (5)	0	2 (9.1)	0.429
**Trifecta – no. (%)**	**51 (60.7)**	**3 (17.6)**	**13 (65)**	**23 (92)**	**12 (54.5)**	**< 0.001**
**Conversion to TUR-P – n (%)**	**10 (11.9)**	**9 (52.9)**	**0**	**0**	**1 (4.5)**	**< 0.001**
**Lobe technique**						**< 0.001**
1	1 (1.2)	0	0	0	1 (4.5)	
2	38 (45.8)	3 (17.6)	0	21 (87.5)	14 (63.6)	
3	44 (53)	14 (82.4)	20 (100)	3 (12.5)	7 (31.8)	
Prostate biopsy – n (%)	23 (28)	7 (41.2)	3 (15)	11 (47.8)	2 (9.1)	0.008
Stress/Intraoperative complications – n (%)	11 (13.3)	2 (11.8)	1 (5.0)	0	8 (38.1)	0.071

At six-weeks postoperative review, the median IPSS was 4 (IQR: 2–6) points and the median Qmax 21.4 (IQR: 13.2–28) ml/sec. Overall, the IPSS improved by 12.5 (8–16) points (p value: 0.566) and the Qmax by 208% (109.8-266.7) (p value: 0.411).The readmission rate was 17.9% with a median re-hospitalization of 3 (IQR: 2–4) days. A total of 18% patients experienced sequelae based on the Clavien Dindo classification. Most complications were, however, minor. All follow-up data are available in Table 3.

**Table 3:** Outcome after six weeks. Values are presented as mean ± standard deviation or median with interquartile range and n (%)

	Total	1	2	3	4	*p*-value
Number of cases	84	17	20	25	22	
IPSS	4 (2–6)	6.5 (6–7)	4.5 (3–6)	5.5 (4–7)	3 (2–5)	0.091
IPSS improvement	12.5 (8–16)	8 (8–8)	16 (16–16)	15 (4.5–19.5)	12.5 (6-15-5)	0.566
Qmax (mL/sec)	21.4 (13.2–28)	35.5 (29.2–47.3)	22.2 (8.5–27)	18.5 (12.5–23.4)	20.5 (16–27)	0.060
Qmax improvement in %	208.3 (109.8-266.7)	n/a	558.5 (558.5-558.5)	139 (98–289)	208.3 (109.8-266.7)	0.411
**Readmission**,** n (%)**	**15 (17.9)**	**7 (41.2)**	**6 (30)**	**1 (4)**	**1 (4.5)**	**0.002**
**Clavien Dindo Complications**,** n (%)**						**0.003**
1	1 (1.2)	0	0	0	1 (4.5)	
2	10 (11.9)	5 (29.4)	5 (25)	0	0	
3	3 (3.6)	1 (5.9)	1 (5.0)	1 (4.0)	0	
4	1 (1.2)	1 (5.9)	0	0	0	

IPSS = International Prostate Symptom score

**Fig. 1:**
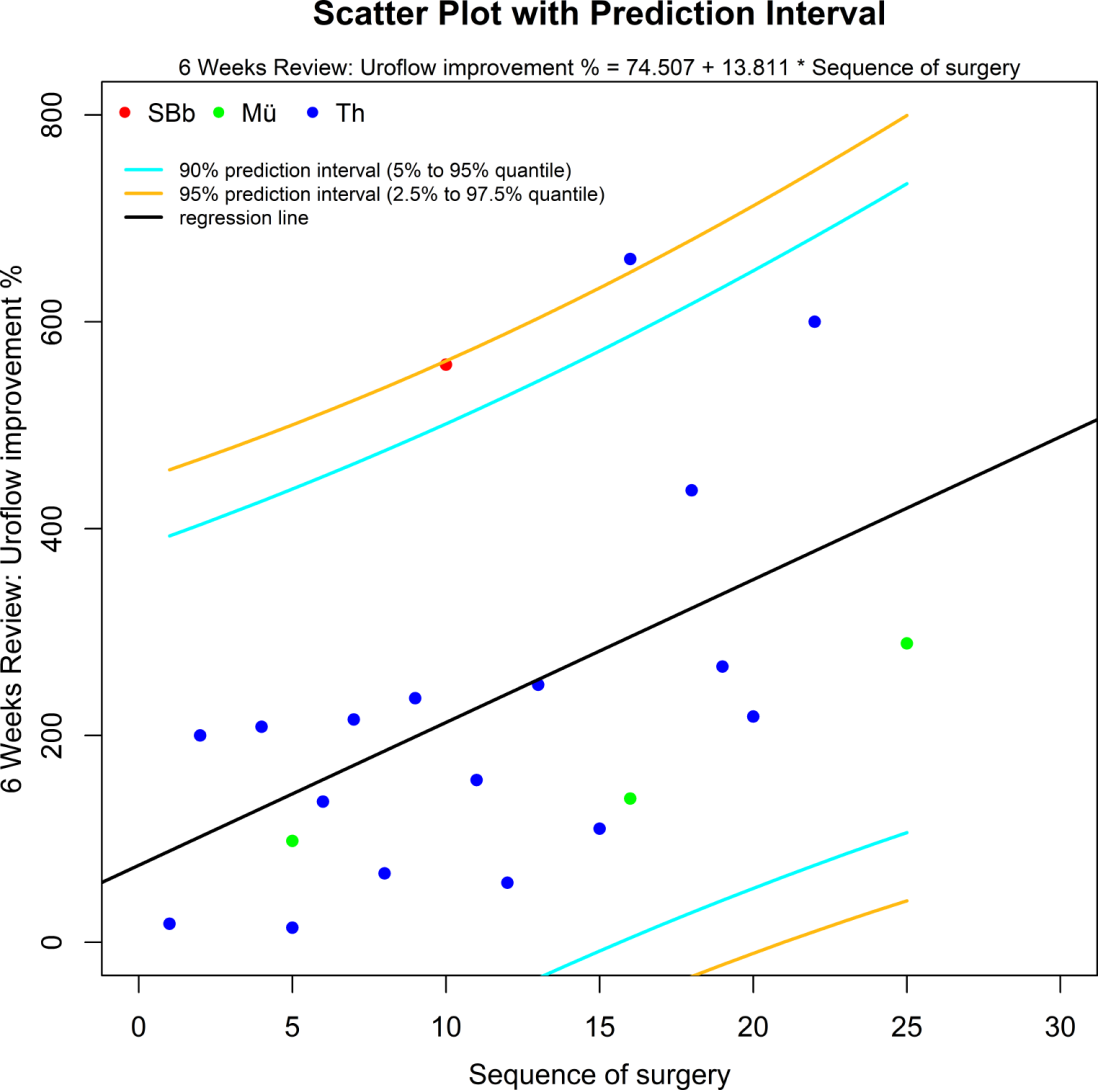
Linear correlation of learning curve with Uroflow improvement for the different centres

Furthermore, we conducted a linear regression analysis (Table 4). A statistically significant increase (*p* = 0.018) in terms of improvement of Uroflow could be shown (Fig. 1) in correlation with the sequence of surgery over all centres. The model formula is “Uroflow improvement % = 74.507 + 13.811* sequence of surgery”. Moreover, a linear improvement of Uroflow could also be shown for one single center (Surgeon 4, *p* = 0.005).

**Table 4:** Linear regression analysis for Uroflow improvement

	Unstandardized coefficents		Standardized coefficents	Test statistic	*p*-value
	B	Std. Error	Beta		22
Constant	74.507	71.504		1.042	0.310
**Sequence of surgery**	13.811	5.339	0.510	2.587	**0.018**

However, no statistical significance could be demonstrated for operating time (*p* = 0.842), hemoglobin drop, resected prostate volume in % (*p* = 0.452) or complications (intraoperative, postoperative) (*p* = 0.941) with the sequence of cases.

## Discussion

The present multiinstitutional, multinational, retrospective cohort study showed that BipolEP can be safely performed by surgeons with limited experience with the technique providing albeit a significant improvement in voiding symptoms. Importantly, all surgeons presented very good short-term outcomes. In particular, the volume of the enucleated tissue, the operative time, the TRIFECTA outcomes, as well as the low number and severity of perioperative complications indicate that BipolEP is feasible and effective even among surgeons at their very early learning curve. A statistical significance was demonstrated for Uroflow-improvement after 6 weeks with the sequence of cases. However, no significance was shown regarding operating time or complication rate. Yet, it must be highlighted that the Uroflow-improvement might be biased by the high conversion rate to TUR-P, especially in one surgeon.

Studies that look into the learning curve of BipolEP are scarce. Xiong et al. in a retrospective cohort of the first consecutive 100 cases with a mean patient age of 69 years demonstrated that BipolEP is a safe and reproducible procedure. The mean prostate volume was 75 ± 27 ml preoperatively and a total of 43 ± 20 ml were removed in 118 min which very much resembles the results of our study. In the first 100 cases, 17 patients required partial conversion to bipolar TUR-P, whereas Qmax, IPSS, quality of live and post void residual significantly improved up to 6 months after the operation. Perioperative complications were rare and, in most cases, minor. Importantly, the authors indicate that major perioperative outcomes (TRIFECTA, functional outcomes and perioperative complications) reached a plateau after 50 cases, suggesting that the learning curve of BipolEP is about 50 cases [9].

These findings have been further corroborated by Hirasawa et al. [10]. In this retrospective study with a two-year follow up of a single surgeon’s experience in 603 consecutive patient undergoing BipolEP with a mean age of 70 years, the authors demonstrated that BipolEP represents a safe and effective surgical procedure. In particular, the mean operative time was 58 ± 1.1 min and the mean prostatic volume 31 ± 0.7 ml, leading to a BipolEP efficiency of 0.54 ± 0.01 ml/min. The learning curve analysis for BipolEP demonstrated a stabilization in the BipolEP efficiency at 46–55 cases, suggesting that mastering BipolEP comes with a steep learning curve and that the efficiency of BipolEP improves markedly when the level of experience exceeds 50 cases. At the two year follow-up, a significant improvement in Qmax (27 ± 1.3 ml/sec, *p* < 0.001), IPSS (4 ± 0.19, *p* < 0.001), and quality of live (1 ± 0.06, *p* < 0.001) was demonstrated compared to the preoperative values. Of note, the reported short- and long-term complications of BipolEP were rare and, in most cases, minor.

Learning curve estimations of all surgical techniques due to BPH have been previously attempted in the literature [11]. Although TUR-P is considered easier to learn, it might be, still, challenging for a beginner, presenting a learning curve of about 50 cases. Similarly, HolEP and other laser enucleation techniques require a learning cuve of about 50 cases among surgeons having already mastered TUR-P and other endourological procedures for BPO [6]. Nevertheless, it should be noted that more than 100 cases are needed to achieve optimal outcomes with minimal complications [12]. Of note, water vapor thermal therapy (Rezum^R^) among the minimally invasive surgical treatments (MIST) for BPO needs only a minimal learning curve of a handful cases but is not considered cavitating surgery like BipolEp [13, 14]. Overall, all BPO surgical techniques that involve tissue resection are not devoid of complications even in the hands of highly experienced surgeons, indicating that the use of simulator technologies, surgical observation and proctorship into training pathways is mandatory to improve short- and long-term outcomes after surgery [15].

We believe that our study, by showing a very encouraging rate of convertion to TUR-P (11.9%) which by the way was heavily skewed by the results of one surgeon who has an almost 50% conversion rate to TUR-P, has provided some evidence to consider 20 cases as the minimum number of cases to become competent in performing BipolEp.

It should be highlighted that the present study displays certain limitations relevant to its retrospective design, short follow-up and relatively small number of included surgeons and patients. Based on the previous notion, our study is underpowered to demonstrate statistical significance in further perioperative outcomes (operating time, complications). The reason that one surgeon, who also was the youngest, was having so many conversion to TUR-P (> 52%) can not be easily explained: Extensive experience in transurethral surgery might be mandatory in learning enucleation, which can be found in other publications [16]. It can only be assumed, that in our surgeon-cohort there is different surgical experience. Moreover, the small number of participants, and the fact that some baseline characteristics and outcomes were missing did not allow us to perform additional analyses. Of note, important outcomes such as the post void residual, the enucleation and morcellation time, erectile and ejaculation function, the number of cases detected with prostate cancer, the infection rates and the need for medications and adjunct treatment after discharge were not systematically evaluated for the present analysis. Importantly, we did not assess the severity and the type of urinary incontinence. It should be also stressed that it was beyond the present study to evaluate the long-term outcomes of surgeons starting to learn BipolEP.

Another potential limitation could be related to the absence of the surgeon’s point of view on the learning curve of the procedure, the level of difficulty of the different steps and the threshold for conversion to TUR-P which was not pre-defined and left to the surgeon’s discretion.

To summarize, it can be stated that there is a variability on the level of difficulty encountred among surgeons starting to perform BipolEP, the procedure itself it not easy to learn but has the big advantage of rapid convertion to standard TUR-P with minimal added morbidity to the patient.

## Conclusion

The present study demonstrates that BipolEp can be successfully performed with good outcomes and an acceptable safety profile following a minimum learning curve of 20 cases in the hands of surgeons with significant experience in transurethral resection. Surgeons starting to learn BipolEP can expect to be able to achieve a linear improvement in Uroflow at the six-week postoperative evaluation after 20 consecutive cases. Even when encountering problems during the early learning curve, BipolEp provides the surgeon with a safe rapid solution by convertion to standard TUR-P although this will only be necessary for a small subset of patients.

## Data Availability

The datasets generated and/or analysed during the current study are not publicly available due to no open access to Paracelsus Medical University medical records data base, but are available from the corresponding author on reasonable request.
